# Histology and immunofluorescent study of the pancreas in lovebird (*Agapornis personatus*)

**DOI:** 10.1002/vms3.1394

**Published:** 2024-03-09

**Authors:** Nader Goodarzi, Ayda Bashiri

**Affiliations:** ^1^ Department of Basic Sciences and Pathobiology, Faculty of Veterinary Medicine Razi University Kermanshah Iran; ^2^ Faculty of Veterinary Medicine, Islamic Azad University Sanandaj Branch Sanandaj Iran

**Keywords:** alpha cell, beta cell, bird, insulin, islet of Langerhans

## Abstract

**Background:**

Lovebird (*Agapornis personatus*) is a monotypic species of bird of the lovebird genus in the parrot family Psittaculidae and order *Psittaciformes*.

**Objectives:**

The present study was designed to investigate the histology and immunohistochemistry of the pancreas in the lovebird.

**Methods:**

Totally, three adult birds were used. The pancreas was assessed using histological and immunofluorescent staining to detect insulin, glucagon, somatostatin, pancreatic polypeptide (PP) and neuropeptide Y (NY).

**Results:**

The exocrine pancreas was composed of pyramidal acinar cells with zymogen granules at the apical cytoplasm. The endocrine pancreas was identified as large alpha, small beta and mixed islets of Langerhans. No intercalated duct was observed. Alpha cells with a density of 28.55% were the most numerous cell type, which were populated throughout the large islets, especially at the periphery. The beta cells with a density of 15.78% were accumulated mostly at the periphery of islets. The delta cells exhibited 17.81% intensity. Despite their lower density, the distribution of delta cells was like that of A cells throughout the islets. PP and NY cells were distinguished with densities of 14.69% and 20.63%, respectively.

**Conclusions:**

Although the arrangement of acinar cells, ductal systems and endocrine islets reflects patterns observed in various avian species, the absence of intercalated duct, the presence of three types of Langerhans islets as alpha, beta and mixed islets and the high expression of NY in the islets were some unique features observed in the current study. These findings contribute to the broader understanding of avian pancreas histology.

## Graphical abstract

The endocrine pancreas was identified as large alpha islets of Langerhans, small beta islets and mixed islets. The intercalated duct was absent. Alpha cells with a density of 28.55% were the most numerous cell type; however, PP cells with densities of 14.69% were the least cells.

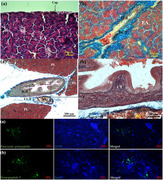

## 1 INTRODUCTION

It is well known that the pancreas of animals, as an important gland of the digestive system, is constituted of two distinct portions: the exocrine part, which releases digestion‐related enzymes, and the endocrine portion, which is involved in the secretion of regulatory hormones, including insulin, glucagon and somatostatin (Das et al., 2013; Rawdon, 1998).

The exocrine pancreas is composed of acinar cells, which are actively involved in producing and releasing secretory zymogen granules. These granules emptied through an extensive ductal system into the duodenal lumen (Banks, 1993). The endocrine pancreas is scattered in the exocrine part as an islet of Langerhans. The islets can be subdivided into three types: alpha islets, beta islets and mixed islets (Simsek et al., 2008).

Comparing the pancreas of the mammalian and avian species reveals that there are some prominent differences between them. In contrast to mammals, whose pancreas is composed of two lobes, the avian pancreas is composed of two to four separate lobes (Rawdon, 1998). Furthermore, from a developmental viewpoint, in contrast to the embryonic pancreatic tissue of mammals, which develops from two duodenal buds, the avian pancreas develops from three buds of the duodenum, including dorsal and ventral evagination (Simsek et al., 2008).

Previously, the anatomical features and histological structure of the pancreas have been investigated in some avian species (Al‐Haaik, 2019; Al‐Khakani et al., 2019; Beheiry et al., 2018; Hamodi et al., 2013; Mahmood et al., 2022; Nascimento et al., 2007; Palmieri & Shivaprasad, 2014; Saadatfar & Asadian, 2009; Simsek et al., 2008). According to the reported results, the macroscopic and microscopic characteristics of the pancreas can be widely different between avian species regarding its lobe number, type and distribution of islets, type of endocrine cells in islets and pancreatic duct number and structure. For instance, it has been reported that, despite other avian species, the pancreas in the cattle egret is not lobulated (Yehia et al., 2021). Or in microscopic structure, usually the ductal system begins with centroacinar cell, however, it was reported that this cell was absent in the Guinea fowl, common gull (Hamodi et al., 2013) and mynah (Saadatfar & Asadian, 2009). *Agapornis personatus*, which is known as lovebird, is a monotypic species of bird of the lovebird genus in the parrot family Psittaculidae and order *Psittaciformes*. This bird is distributed originally in the Arusha Region of Tanzania and has been introduced to Burundi and Kenya. The lovebirds eat primarily seeds of trees and grasses, including Acacia seeds, millet and sorghum, mixed vegetable and some fruits (Appleyard, 2001; Juniper and Parr, 1998).

Therefore, this study was conducted to investigate the anatomical and histological structure as well as the immunohistochemical properties of the pancreas in the love bird (*A. personatus*). The obtained results are compared and discussed with previous observations of other domestic and wild avian species.

## 2 MATERIALS AND METHODS

### 2.1 Sample collection and preparation

In the present work, three dead birds with an average weight of 135 ± 7 g were used. The subjects were dead due to diseases unrelated to the digestive system. The birds were transformed into the dissection room immediately after death. The abdominal cavity was opened, and the viscera were detected. The position of the pancreas in the duodenal loop, its lobes and related excretory ducts were dissected and identified.

### 2.2 Histological section preparation

For preparing histological sections, tissue samples were fixed in 10% neutral buffered formalin. After 1 week, the fixed samples were dehydrated in an ascending series of alcohols, cleared in xylene and embedded in paraffin wax. The paraffin blocks were cut into 6‐μm sections using a rotatory microtome. The obtained sections were stained routinely with haematoxylin and eosin for studying general histological details, periodic acid–Schiff (PAS) for detecting carbohydrate and Masson's trichrome for staining connective tissue (CT) contents (Abumandour & Kandyel, 2020; El‐Mansi et al., 2020).

### 2.3 Immunofluorescent procedure

Immunofluorescence staining for insulin, glucagon, somatostatin, neuropeptide Y (NY) and pancreatic polypeptide (PP) markers was performed on formalin‐fixed and paraffin‐embedded tissues. After deparaffinizing in xylene (three changes for 5 min each) and rehydrating with ethanol (two changes in 100% ethanol, two changes in 95% ethanol and one change in 70% ethanol, for 5 min each), the obtained sections were incubated in TBS 1× (T5912‐Sigma) (95°C for 20 min). The slides were washed with PBS three times at a 5 min interval. Triton 0.3% was added to increase the permeability of the cell membrane. After washing with PBS (Phosphate‐buffered saline), the samples were treated with 10% goat serum for 45 min. As a primary antibody, glucagon polyclonal rabbit antibody (Cat no. orb539598, Biorbyt LLC), insulin polyclonal rabbit antibody (Cat no. orb536255, Biorbyt LLC), somatostatin polyclonal rabbit antibody (Cat no. GTX133119, GeneTex), PP polyclonal rabbit antibody (Cat no. 15493‐1‐AP, Proteintech) and NY polyclonal rabbit antibody (Cat no. orb11154, Biorbyt LLC) were diluted 1:100 in PBS and applied overnight at 4°C. After washing with PBS, A goat anti‐rabbit IgG (H + L) (Cat no. orb688925, Biorbyt LLC) was used as a secondary antibody with a dilution of 1–150 and incubated at 37°C for 90 min. The sample was transferred from the incubator to a dark room, and after three washes, DAPI (D9542‐Sigma) was added to them. After 20 min, the slides were washed with PBS and analysed with a fluorescent microscope. The intensity of staining was calculated using ImageJ software. Totally, two slides from each sample and five fields of view in each section were analysed.

## 3 RESULTS

### 3.1 Histological observations

**FIGURE 1:**
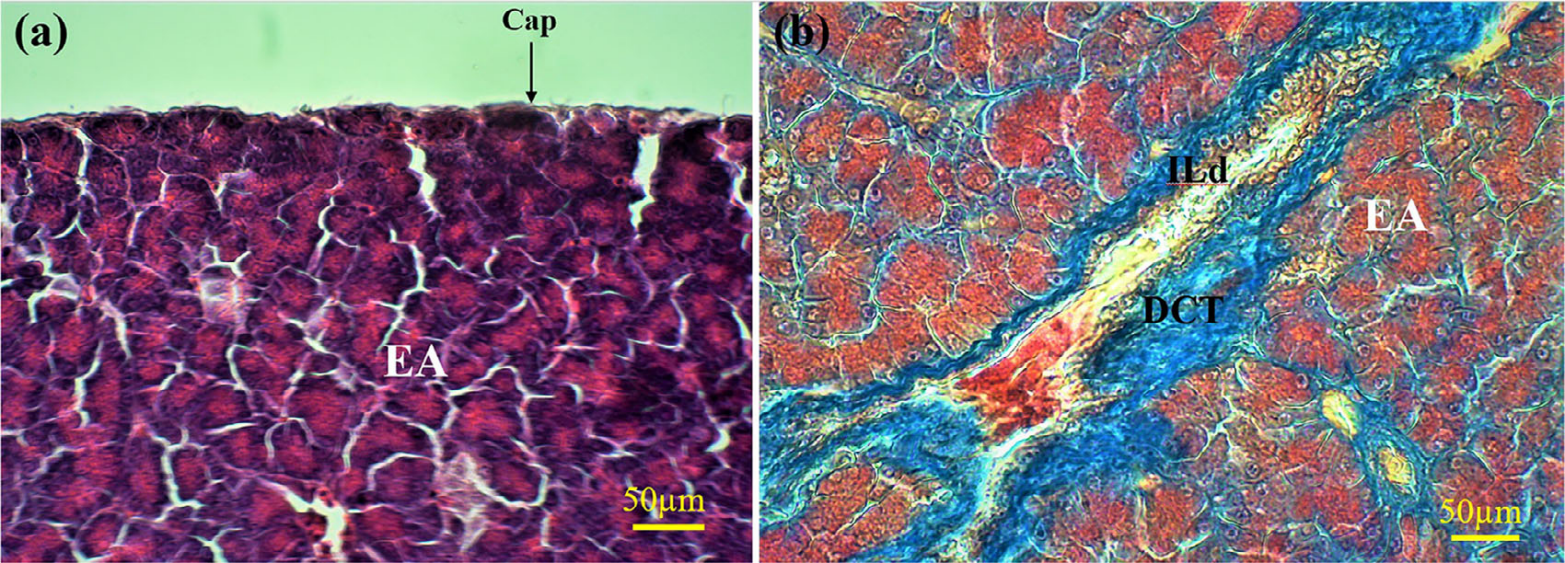
Micrograph of the exocrine pancreas in lovebird. Part (a) shows capsule of the pancreas (Cap) and exocrine acini (EA) using haematoxylin and eosin (H&E) staining. Part (b) shows an intralobular duct (ILD) surrounded by dense connective tissue (DCT) between several EA using Masson's trichrome staining.

**FIGURE 2:**
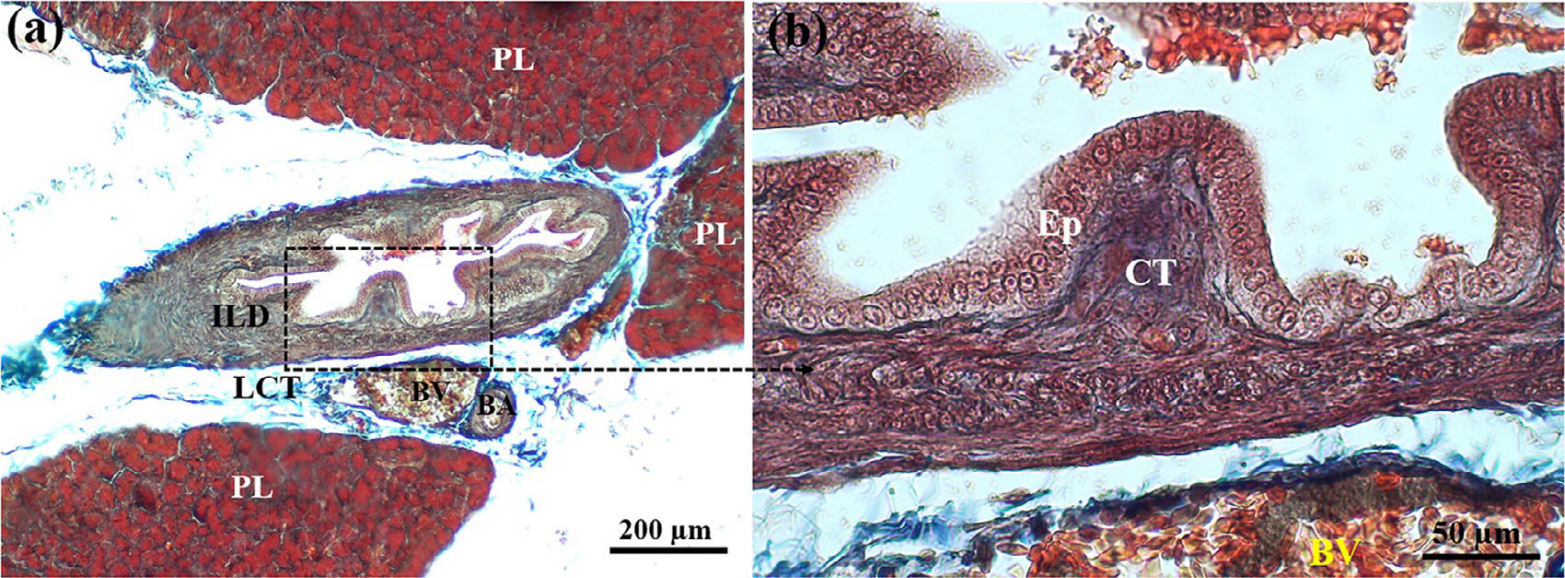
Micrograph of the exocrine pancreas in lovebird. Part (a) shows an interlobular duct (ILD) between three pancreatic lobes (PL), which was surrounded by loose connective tissue (LCT), blood vein (BV) and blood artery (BA). Part (b) shows the black box in (a) at higher magnification. Note the short simple columnar epithelium (Epi) and connective tissue (CT) in the wall of the interlobular duct and its folding (haematoxylin and eosin [H&E] staining).

The pancreas of the lovebird was covered by a thin CT capsule. The capsule sent several septa into the pancreatic parenchyma and divided the pancreatic tissue into numerous lobules (Figure 1a). The lobules of the exocrine pancreas were composed of acinus units with different shapes and sizes. The shape of the acini varied from spherical or round to irregular. The cells of the acini were pyramidal in shape. The apical cytoplasm was eosinophilic due to the accumulation of zymogen granules. The basal cytoplasm was dark basophilia and contained the nucleus. The ductal system of the pancreas was composed of centroacinar cells and intralobular and interlobular ducts (ILDs). No intercalated duct was seen. The epithelium of ILDs was simple and cuboidal (Figure 1b); however, the interlobular ones were composed of short simple columnar epitheliums (Figure 2a,b).

**FIGURE 3:**
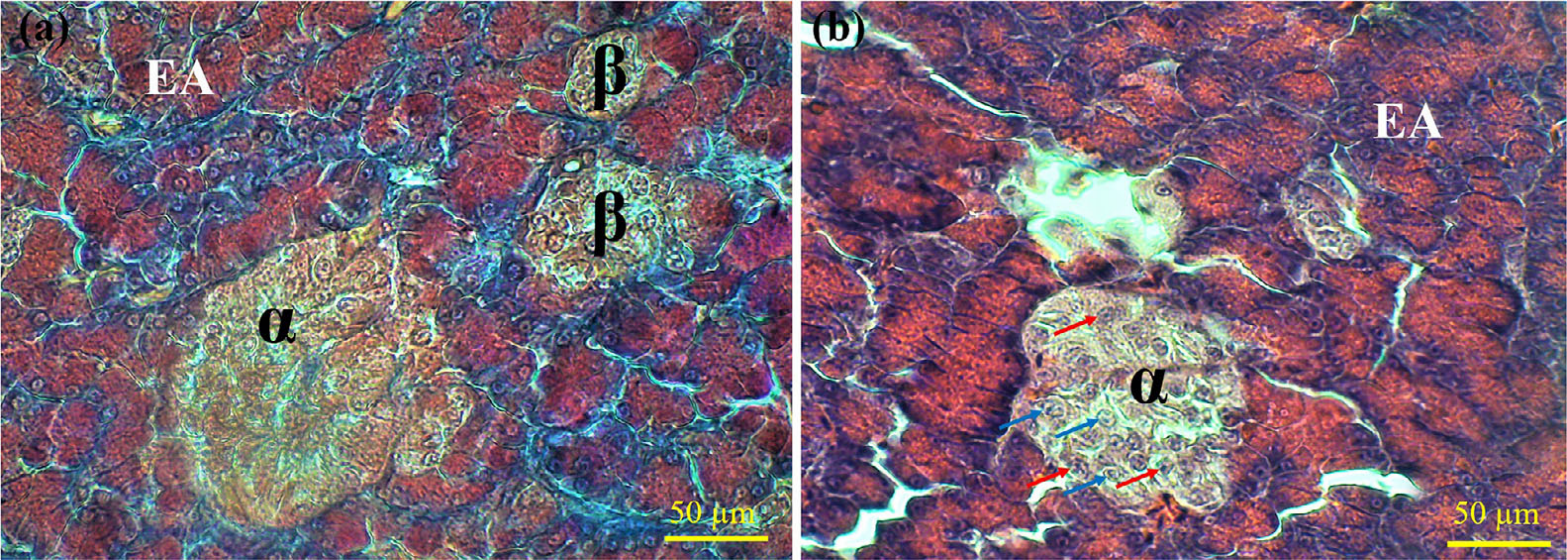
Micrograph of the endocrine pancreas in lovebird. Part (a) shows a large alpha (*α*) and two small beta (*β*) islets using Masson's trichrome staining. Part (b) shows a large alpha (*α*) islet using haematoxylin and eosin (H&E) staining. EA: exocrine acini, blue arrows: alpha cells, red arrows: beta cells.

**FIGURE 4:**
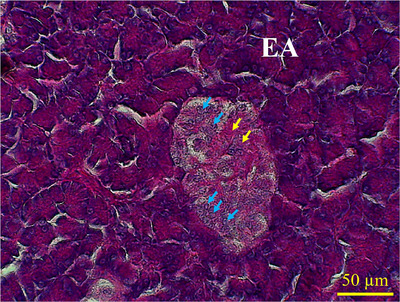
Micrograph of pancreatic parenchyma in lovebird showing a beta islet. Yellow arrows indicate eosinophilic or purple‐stained cytoplasm of beta cells with, blue arrows indicate alpha cell's nucleus at the periphery of islet. Periodic acid–Schiff (PAS) staining.

The endocrine islets of Langerhans were detected as aggregations of cells with different sizes and shapes distributed throughout the exocrine parenchyma. The shape of islets varied from spherical to ovoid, and some islets had irregular shapes. The size of islets varies significantly, ranging from very small to quite large. The alpha islets were larger than the beta ones. The alpha cells, which secrete glucagon, were more concentrated on the periphery of the islets; similarly, the beta cells showed a peripherally located population (Figure 3a,b). The blood capillary cells were in direct contact with the islet. The beta cells of islets showed purple coloration against PAS staining, which indicated their glycogen contents (Figure 4).

### 3.2 Immunofluorescent observations

**FIGURE 5:**
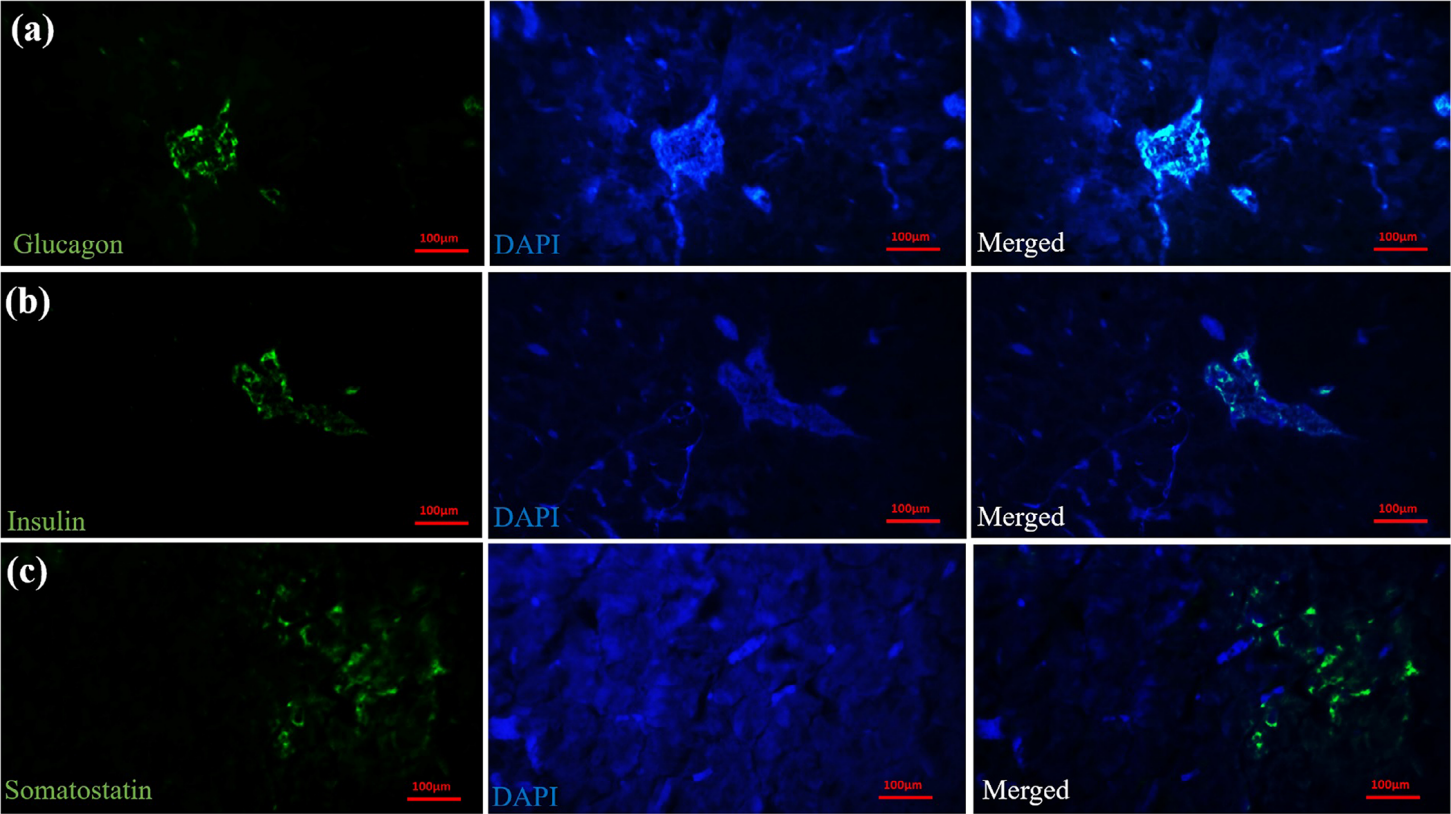
Immunofluorescent staining for detecting (a) glucagon, (b) insulin and (c) somatostatin immune‐positive cells (green stained cells) in the pancreas of lovebird. The left panel shows the desired antibody in green staining. The middle panel is DAPI staining of cell nucleus, and the right panel is merged of the left and middle panels.

**FIGURE 6:**
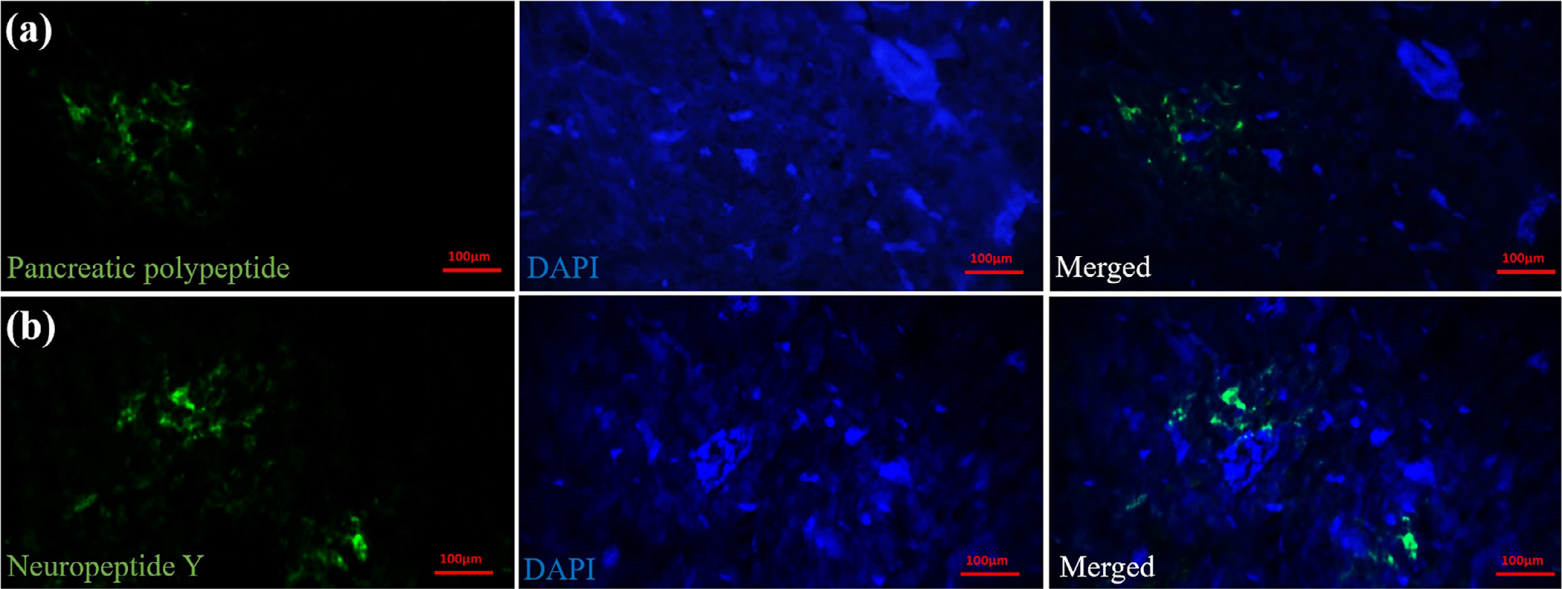
Immunofluorescent staining for detecting (a) pancreatic polypeptide and (b) neuropeptide Y‐immune‐positive cells (green stained cells) in the pancreas of lovebird. The left panel shows the desired antibody in green stain. The middle panel is DAPI staining of cell nucleus, and the right panel is merged of the left and middle panels.

All five types of endocrine cells were distinguished in the islets of Langerhans. The alpha cells (glucagon‐secreting cells) with a peripheral location density of 28.55% ± 3.67% were the most numerous cells in the islets (Figure 5). The beta cells (insulin‐secreting cells) were concentrated at the periphery of the islets with a density of 15.75% ± 6.11% (Figure 5). The delta cells (somatostatin‐secreting cells) presented a density of 17.81% ± 3.18% (Figure 5). PP cells showed a density of 14.69% ± 2.69%, and NY cells exhibited a density of 20.63% ± 1.94% (Figure 6). Overall, three types of endocrine islets were distinguished: large alpha islets, which were mainly composed of alpha and delta cells; small beta islets, composed of beta cells; and mixed islets, which contained alpha and beta cells. The percentage of expression and distribution pattern of endocrine cells is presented in the Table 1.

**TABLE 1:** The percentage of expression and distribution pattern of endocrine cells in the pancreas of lovebird (*Agapornis personatus*).

Cell types	Percentage of expression	Distribution pattern
Alpha cells	28.55% ± 3.67%	Periphery of the islet
Beta cells	15.75% ± 6.11%	Periphery of the islet
Delta cells	17.81% ± 3.18%	Throughout the islet
PP cells	14.69% ± 2.69%	Throughout the islet
NY cells	20.63% ± 1.94%	Throughout the islet

Abbreviations: NY, neuropeptide Y; PP, pancreatic polypeptide.

## 4 DISCUSSION

The histological examination of the lovebird pancreas has unveiled some structural characteristics and endocrine cell distributions within the islets of Langerhans. This discussion will delve deeper into the implications of these observed histological features, compare them to existing literature and unravel their potential functional significance.

### 4.1 Histological architecture

The architecture of the lovebird pancreas stands out as a harmonious blend of specialized compartments. The enveloping CT capsule, along with the partitioning of the exocrine pancreatic tissue into lobules, is consistent with the typical organization previously documented in domestic and wild birds (Al‐Haaik, 2019; Beheiry et al., 2018; Gulmez et al., 2004; Hamodi et al., 2013; Helmy et al., 2018; Saadatfar & Asadian, 2009; Saadatfar et al., 2011; Simsek et al., 2008). The presence of acinar units, each having diverse shapes and sizes, reflects the secretion of known digestive enzymes (Watanabe et al., 1975). Regarding the shape of acini, according to our observation, acinar unites were polygonal to round; they were reported to have a columnar appearance in pigeons (Mobini, 2013), turkeys (Mobini, 2013) and gooses (Gulmez et al., 2004).

Of particular interest is the intriguing absence of intercalated ducts, a distinctive feature not commonly reported in avian pancreas histology. This remarkable finding could hint at unique physiological adaptations in lovebirds, possibly related to specific digestive processes or secretory mechanisms. The ILD was covered by simple cuboidal epithelium in the current work, whereas the ILD epithelium was simple columnar type. This finding about the ILDs comes in line with other descriptions in the duck (Mahmood et al., 2022), goose (Gulmez et al., 2004), turkey (Suri et al., 2022) and kestrel (Al‐Haaik, 2019). The distinctive characteristics of intralobular and ILDs further emphasize their vital role in modifying and transporting pancreatic secretions before releasing them into the duodenum.

### 4.2 Endocrine islets of Langerhans

The distribution of endocrine islets within the exocrine tissue reveals a fascinating heterogeneity. The observation of islets with varying sizes, ranging from very small to very large, aligns with the hypothesis that islet size may be intricately linked to functional specialization and hormonal demands. The characteristics of alpha and beta islets offer compelling insights into potential adaptations. The peripheral concentration of alpha cells in the larger alpha islets, which are responsible for glucagon secretion, might signify their pivotal role in swiftly responding to glucose imbalances during periods of heightened metabolic needs or stress. Similarly, the peripheral positioning of beta cells, responsible for insulin secretion, aligns with their primary role in orchestrating glucose homeostasis (Kimmel et al., 1968; Watanabe et al., 1975). The intimate proximity of islet cells to adjacent blood capillaries accentuates the significance of rapid and efficient hormone release into circulation.

### 4.3 Immunofluorescent staining

Immunofluorescent analyses afford us a quantitative understanding of the densities of different endocrine cell types within the islets. The higher prevalence of alpha cells and beta cells compared to other endocrine cells underscores their pivotal roles in orchestrating the concentration of glucagon and insulin in glucose regulation (Kimmel et al., 1968). The presence of additional endocrine cell types, such as delta, PP and NY cells, reveals the intricacy of the endocrine pancreas's regulatory functions and hints at a broader involvement in a myriad of physiological processes.

Regarding the position of alpha cells, the present finding was like those reported previously in goose (Gulmez et al., 2004), Japanese quail (Simsek et al., 2008), ostrich (Tarakci et al., 2007) and long‐legged buzzard (Bayrakdar et al., 2011). However, the present observations revealed that beta cells were more concentrated at the periphery of the islets. This finding was in contrast to those described in other avian species (Gulmez et al., 2004; Mobini, 2013; Sismek & Alabay, 2008) and most mammalian species (Kim et al., 2009; Steiner et al., 2010).

Avian pancreas can contain three types of islets: alpha, beta and mixed islets (Simsek et al., 2008). As the present findings revealed, three types of islets were distinguished in the endocrine pancreas of the lovebird. This result contrasts with the studies in the pigeon (Mobini, 2013) and geese (Gulmez et al., 2004), which reported the absence of mixed islets. However, the islets in the common gull were reported to be only of mixed type (Hamodi et al., 2013).

The present finding indicates an even distribution of delta cells throughout the islets. This observation comes in line with those reported in sparrow hawks (Kara et al., 2014) and gooses (Gulmez et al., 2004). Furthermore, the higher concentration of delta cells as compared to beta cells was an interesting finding. Physiological studies revealed that the insulin content of the avian pancreas can be 1/7–1/10 of the mammalian species values. On the other hand, the plasma and pancreas concentrations of glucagon are 10 times those of mammals (Kimmel et al., 1968; Watanabe et al., 1997).

These observations suggest a pronounced influence of glucagon and somatostatin, compared to insulin, on the morphological aspects of glucose metabolism in avian species (Trimble & Renold, 1981; Unger et al., 1978).

In the present study, NY‐secreting cells were the most populated endocrine cells after the alpha cells. However, the PP‐secreting cells showed the lowest percentage. The presence of the NY hormone has been explored in both avian embryos and adults. Therefore, concurrent expression of NY with glucagon, insulin and somatostatin in embryonic and adult stages suggests the likelihood of multi‐hormonal progenitor cells in the avian pancreas (Lucini, 2000). It is noteworthy that avian PP secretion can be mediated by dual complementary alpha‐ and beta‐adrenoreceptor mechanisms (Meglasson and Hazelwood, 1983). PP is a 36‐amino‐acid secretory protein that is predominantly produced by the pancreas that affects the secretion of pancreatic enzymes, electrolytes and water. The avian PP has some different properties as compared with mammalian PP. Blundell et al. (1981) investigated the molecular structure of the avian PP using X‐ray and described that PP molecule has several general features in common with the pancreatic insulin and glucagon. All three hormones have complex mechanisms for self‐association. Like insulin, avian PP seems to have a stable monomeric structure, but its biological activity seems to depend on the more flexible COOH‐terminal region, analogous to the flexible NH_2_‐terminal region of glucagon.

## 5 CONCLUSION

The findings of this study within the landscape of avian pancreas histology reveal some specific properties. The arrangement of acinar cells, ductal systems and endocrine islets echoes patterns observed in various avian species, offering insights into the evolutionary conservation of these features. However, the absence of intercalated ducts, the presence of three types of Langerhans islets (alpha, beta and mixed islets) and the high expression of NY in the islets were some unique features observed in the current study. The presence of diverse endocrine cell types invites further investigation into their contributions to lovebird physiology, potentially extending beyond glucose regulation. These findings contribute to the broader understanding of avian pancreas histology and offer some insights into potential functional adaptations.

## AUTHOR CONTRIBUTIONS

Ayda Bashiri contributed to sample preparation and laboratory practices; Nader Goodarzi interpreted the results and wrote the manuscript draft.

## CONFLICT OF INTEREST STATEMENT

The authors declare no conflicts of interest.

### ETHICS STATEMENT

None.

### PEER REVIEW

The peer review history for this article is available at https://www.webofscience.com/api/gateway/wos/peer-review/10.1002/vms3.1394.

## Data Availability

Research data are not shared.

## References

[vms31394-bib-0001] Abumandour, M. M. A. , & Kandyel, R. M. (2020). Age‐related ultrastructural features of the tongue of the rock pigeon *Columba livia* dakhlae in different three age stages (young, mature, and adult) captured from Egypt. Microscopy Research and Technique, 83, 118–132. 31971320 10.1002/jemt.23394

[vms31394-bib-0002] Al‐Haaik, A. G. (2019). A gross anatomical and histological study of pancreas in adult Kestrel (*Falco tinnunculus*). Iraqi Journal of Veterinary Sciences, 33(2), 175–180.

[vms31394-bib-0003] Al‐Khakani, S. S. A. , Zabiba, I. J. M. , Al‐zubaidi, K. H. , & Al‐alwany, E. A. H. (2019). Morphometrical and histochemical foundation of pancreas and ductal system in white‐eared bulbul (*Pycnonotus leucotis*). Iraqi Journal of Veterinary Sciences, 33(1), 99–104.

[vms31394-bib-0004] Appleyard, V. (2001). The lovebird handbook. Barron's Educational Series. Inc.

[vms31394-bib-0023] Banks, W. J. (1993). Applied Veterinary Histology. 3rd Ed, Mosby‐Year book. Inc.

[vms31394-bib-0030] Bayrakdar, A. , Yaman, M. , Atalar, O. , Gencer, T. B. , & Ceribasi, S. (2011). Distribution of neuropeptides in endocrine and exocrine pancreas of long‐legged buzzard (Buteo rufinus): An immunohistochemical study. Regulatory Peptids, 166, 121–127. 10.1016/j.regpep.2010.10.007 20959123

[vms31394-bib-0005] Beheiry, R. R. , Abdel‐Raheem, W. A. , Balah, A. M. , Salem, H. F. , & Karkit, M. W. (2018). Morphological, histological and ultrastructural studies on the exocrine pancreas of goose. Beni‐Suef University Journal of Basic and Applied Sciences, 7, 353–358.

[vms31394-bib-0006] Blundell, T. L. , Pitts, J. E. , Tickle, I. J. , Wood, S. P. , & Wu, C. W. (1981). X‐ray analysis (1. 4‐Å resolution) of avian pancreatic polypeptide: Small globular protein hormone. Proceedings of the National Academy of Sciences of the United States of America, 78(7), 4175–4179. 16593056 10.1073/pnas.78.7.4175 PMC319751

[vms31394-bib-0007] Das, A. , Das, R. K. , Parida, S. , Mishra, U. K. , & Solanki, D. (2003). Histomorphological study on pancreas of duck (*Anas boscas*). Indian Journal of Animal Sciences, 73, 598–599.

[vms31394-bib-0008] El‐Mansi, A. , Al‐Kahtani, M. , Abumandour, M. , Ezzat, A. , & El‐Badry, D. (2020). Gross anatomical and ultrastructural characterization of the oropharyngeal cavity of the Egyptian nightjar *Caprimulgus aegyptius*: Functional dietary implications. Ornithological Science, 19, 145–158.

[vms31394-bib-0024] Gulmez, N. , Kocamis, H. , Aslan, S. , & Nazli, M. (2004). Immunohistochemical distribution of cells containing insulin, glucagon and somatostatin in the goose (Anser anser) pancreas. Turkish Journal of Veterinary and Animal Sciences, 28, 403–407.

[vms31394-bib-0009] Hamodi, H. M. , Abed, A. A. , & Taha, A. M. (2013). Comparative anatomical, histological and histochemical study of the pancreas in two species of birds. Research & Review in Biosciences, 8(1), 26–34.

[vms31394-bib-0025] Helmy, S. , & Soliman, M. T. A. (2018). Histological, histochemical and ultra‐structure studies on the Ostrich Pancreas (Struthio camelus). Egyptian Academic Journal of Biological Sciences, 10, 63–77.

[vms31394-bib-0010] Juniper, T. , & Parr, M. (1998). Parrots: A guide to parrots of the world. Yale University Press.

[vms31394-bib-0033] Kara, A. , Tekiner, D. , Simsek, N. , Balkaya, H. , & Ozudogru, Z. (2014). Distribution and Location of Endocrine Cells in the Pancreas of the Sparrowhawk, Accipiter nisus. Kafkas Üniversitesi Veteriner Fakültesi Dergisi, 20(2), 307–312.

[vms31394-bib-0031] Kim, A. , Miller, K. , Jo, J. , Kilimnik, G. , Wojcik, P. , & Hara, M. (2009). Islet architecture: A comparative study. Islets, 1, 129–136. 20606719 10.4161/isl.1.2.9480 PMC2894473

[vms31394-bib-0012] Kimmel, J. R. , Pollock, H. G. , & Hazelwood, R. L. (1968). Isolation and characterization of chicken insulin. Endocrinology, 83, 1323–1330. 4880986 10.1210/endo-83-6-1323

[vms31394-bib-0036] Lucini, C. , Romano, A. , & Castaldo, L. (2000). NPY immunoreactivity in endocrine cells of duck pancreas: an ontogenetic study. Anatomical Record, 259(1), 35–40. 10760741 10.1002/(SICI)1097-0185(20000501)259:1<35::AID-AR4>3.0.CO;2-S

[vms31394-bib-0013] Mahmood, S. K. , Ahmed, N. S. , Sultan, G. A. , & Yousif, M. J. (2022). Histomorphological and carbohydrate histochemical study of the pancreas in native ducks (*Anas Platyrhynchos*). Iraqi Journal of Veterinary Science, 36(4), 1103–1110.

[vms31394-bib-0014] Meglasson, M. D. , & Hazelwood, R. L. (1983). Adrenergic regulation of avian pancreatic polypeptide secretion in vitro. American Journal of Physiology‐Endocrinology and Metabolism, 244, 408–413. 10.1152/ajpendo.1983.244.4.E408 6837734

[vms31394-bib-0015] Mobini, B. (2013). Histochemical and histological studies on the pancreas in mature pigeon (*Columba Livia*). European Journal of Experimental Biology, 3, 148–152.

[vms31394-bib-0016] Nascimento, A. A. , Sales, A. , Cardoso, T. R. D. , Pinheiro, N. L. , & Mendes, R. M. M. (2007). Immunocytochemical study of the distribuition of endocrine cells in the pancreas of the Brazilian sparrow species *Zonotrichia* capensis subtorquata (Swaison, 1837). Brazilian Journal of Biology, 67(4), 735–740. 10.1590/s1519-69842007000400021 18278328

[vms31394-bib-0017] Palmieri, C. , & Shivaprasad, H. L. (2014). An immunohistochemical study of the endocrine pancreas in raptors. Research in Veterinary Science, 97, 587–591. 25468799 10.1016/j.rvsc.2014.10.011

[vms31394-bib-0018] Rawdon, B. B. (1998). Morphogenesis and differentiation of the avian endocrine pancreas, with particular reference to experimental studies on the chick embryo. Microscopy Research & Technique, 43, 292–305. 9849970 10.1002/(SICI)1097-0029(19981115)43:4<292::AID-JEMT3>3.0.CO;2-X

[vms31394-bib-0020] Saadatfar, Z. , & Asadian, M. (2009). anatomy of pancreas in mynah (*Acridotheres tristis*). Journal of Applied Animal Research, 36, 191–193.

[vms31394-bib-0019] Saadatfar, Z. , Asadian, M. , & Alishahi, E. (2011). Structure of pancreas in Palam Dove (*Streptoplia selegalensis*). Iranian Journal of Veterinary Science and Technology, 3(2), 25–32.

[vms31394-bib-0026] Simsek, N. , Ozudogru, Z. , & Alabay, B. (2008). Immunohistochemical on the splenic lobe of the pancreas in young Japanese quail (Coturnix c. japonica). Deutsche Tierärztliche Wochenschrift, 115(5), 189–193. 18547019

[vms31394-bib-0032] Steiner, D. J. , Kim, A. , & Miller, K. (2010). Pancreatic islet plasticity: Interspecies comparison of islet architecture and composition. Islets, 2, 135–145. 20657742 10.4161/isl.2.3.11815 PMC2908252

[vms31394-bib-0028] Suri, S. , Sassan, J. S. , & Khan, A. (2022). Histo‐morphometrical study on the pancreas of Turkey. The Indian Journal of Veterinary Sciences and Biotechnology, 18, 59–62.

[vms31394-bib-0029] Tarakci, B. G. , Yaman, M. , & Bayrakdar, A. (2007). Immunohistochemical study on the endocrine cells in the pancreas of the ostrich (Struthio camelus). Journal of Animal and Veterinary Advances, 6, 693–696.

[vms31394-bib-0034] Trimble, E. R. , & Renold, A. E. (1981). Ventral and dorsal areas of rat pancreas: islet hormone content and secretion. American Journal of Physiology‐ Endocrinology and Metabolism, 240, 422–427. 10.1152/ajpendo.1981.240.4.E422 6111929

[vms31394-bib-0035] Unger, R. H. , Dobbs, R. E. , & Orci, L. (1978). Insulin, glucagons, and somatostatin secretion in the regulation of metabolism. Annual Review of Physiology, 40, 307–343. 10.1146/annurev.ph.40.030178.001515 205166

[vms31394-bib-0027] Watanabe, T. , Paik, Y. K. , & Yasuda, M. (1975). Fine structure of the pancreatic islets in the domestic fowl with special reference to the cell type and secretion. Archivum Histologivum Japonicum, 38, 259–274. 10.1679/aohc1950.38.259 175756

[vms31394-bib-0022] Yehia, O. A. , Ahmed, Y. H. , Elleithy, E. M. M. , Sala, T. F. , & El‐Gharbawy, S. M. S. (2021). Comparative histological, histochemical and ultrastructure studies on the exocrine pancreas of Japanese quail (*Coturnix japonica*) and cattle egret (*Bubulcus ibis*). International Journal of Veterinary Science, 10(2), 107–113.

